# Trophic downgrading of an adaptable carnivore in an urbanising landscape

**DOI:** 10.1038/s41598-023-48868-x

**Published:** 2023-12-07

**Authors:** Gabriella R. M. Leighton, William Froneman, Laurel E. K. Serieys, Jacqueline M. Bishop

**Affiliations:** 1 SARChI Chair in Marine Ecology, Department of Zoology and Entomology, Rhodes University, PO Box 94, Grahamstown, 6140 South Africa; 2 Institute for Communities and Wildlife in Africa, Department of Biological Sciences, University of Cape Town, Rondebosch, Cape Town, 7701 South Africa; 3 Panthera, 8 W 40th St, New York, NY 10018 USA; 4 Cape Leopard Trust, Cape Town, South Africa

**Keywords:** Urban ecology, Stable isotope analysis, Behavioural ecology

## Abstract

Urbanisation critically alters wildlife habitat and resource distribution, leading to shifts in trophic dynamics. The loss of apex predators in human-transformed landscapes can result in changes in the ecological roles of the remaining mesocarnivores. Decreased top–down control together with increased bottom–up forcing through greater availability of anthropogenic foods can result in a predation paradox. Understanding these changes is important for conserving ecological function and biodiversity in rapidly urbanising systems. Here, we use stable isotope analysis to provide insight into longer term changes in trophic position, niche width and overlap of an elusive, medium-sized urban adapter, the caracal (*Caracal caracal*) in and around the city of Cape Town, South Africa. Using fur samples (n = 168) from individuals along a gradient of urbanisation we find that overall caracals have a broad isotopic dietary niche that reflects their large variation in resource use. When accounting for underlying environmental differences, the﻿ intensity of anthropogenic pressure, measured using the Human Footprint Index (HFI), explained variation in both food subsidy use (δ^13^C values) and trophic status (δ^15^N values). The significantly higher δ^13^C values (*P* < 0.01) and lower δ^15^N values (*P* < 0.001) of caracals in more urbanised areas suggest that predator subsidy consumption occurs via predictable, anthropogenic resource subsidies to synanthropic prey. These prey species are predominantly primary consumers, resulting in shifts in diet composition towards lower trophic levels. Further, caracals using areas with higher HFI had narrower isotope niches than those in less impacted areas, likely due to their hyperfocus on a few lower trophic level prey species. This pattern of niche contraction in urban areas is retained when accounting for caracal demographics, including sex and age. The removal of apex predators in human-transformed landscapes together with reliable resource availability, including abundant prey, may paradoxically limit the ecological influence of the remaining predators, and bring about a degree of predator trophic downgrading. The dampening of top–down control, and thus ecosystem regulation, likely points to widespread disruption of trophic dynamics in rapidly developing areas globally.

## Introduction

Urbanisation has significant and often irreversible effects on natural systems, critically altering the structure of both the physical environment and wildlife communities^1^. In remnant wildlife populations these changes can have important ecological and behavioural repercussions, such as shifting foraging strategies or diet composition, which can lead to altered trophic dynamics^2^. In this way, human drivers of environmental change can alter, and sometimes completely disarticulate, long-standing ecological relationships among species that characterise less disturbed systems^1, 3, 4^.

An important consequence of urbanisation is that apex predators are commonly extirpated locally as the human footprint grows and expands^5^. Simultaneously, the influx of resources available to wildlife around cities also alters trophic interactions by favouring adaptable, synanthropic species, thereby bolstering their abundances^6, 7^. Collectively, these changes may result in an increase in medium-rank predators through the process of ‘mesopredator release’^8, 9^. However, due to their smaller size and differing ecologies, medium-rank predators do not always assume the vacated functional roles of apex predators^10^. Nevertheless, the predators that do remain may still drive significantly altered downstream effects through changes in their behaviour brought about by both the removal of top-down pressure, and their adaptation to novel environments^2, 11^. For example, modified behaviours of species in human-transformed areas can influence vulnerability to predation or access to food by disrupting diel patterns and vigilance behaviours^12, 13^. In turn, any resulting shifts in prey communities, bottom-up subsidies, and altered risk landscapes in urban ecosystems can substantially affect contemporary resource use by predators relative to historic patterns^14–16^. In response, the remaining smaller predators may also shift their foraging ecology in unexpected ways that depend on novel prey opportunities and, in doing so, contribute to significant alterations in the trophic ecology of cities.

In natural systems, top predators enforce balance in ecosystems through a combination of top-down and fear effects^11, 17, 18^, and maintaining high overall biodiversity can enhance ecosystem functioning mediated through complex trophic interactions^19^. Understanding how best to conserve ecosystem function in human-impacted areas where top predators are largely lost therefore requires understanding the changes in ecological roles of predators remaining in the system. The analysis of stable isotopes provides a valuable tool to assess changes in the foraging ecology of species in both disturbed and natural systems, and at population and community levels^20, 21^. Importantly, analysis of stable isotopes allows the assessment of tissue-specific integrated signals of carbon and nitrogen assimilated by an individual over time; this is in contrast to the biased snapshot estimates of diet based on what remains undigested (e.g., stomach contents, scat, or prey remains). Stable isotope analysis (SIA) of tissue carbon and nitrogen values can also provide valuable insight into changes in trophic position, niche width and overlap, ontogenetic dietary shifts, and species- and individual-level foraging strategies and habitat use of carnivores in disturbed landscapes^4, 22–24^. While SIA does not give species-level diet information, it can give insight into broader trends. Specifically, higher δ^13^C isotope ratios reflect consumption of prey species with a higher proportion of plants with C_4_ photosynthetic pathways, such as corn (*Zea mays*) and sugarcane (*Saccharum* spp.), which are common in anthropogenic food sources^25^. This is in contrast to lower values associated with predominantly C_3_ plants found in wildland habitats^26, 27^. High δ^15^N isotope ratios indicate consumption of protein-enriched animal prey^21, 22^, denoting trophic status and degree of carnivory (i.e., the position along the continuum from complete herbivory to complete carnivory^28^).

Predators using human-transformed landscapes can benefit from the availability of a diverse and abundant prey base supported by myriad new resources, including fertilisers, water, shelter, refuse, and active provisioning of food. As new sources of food are incorporated into diets, SIA can reveal patterns of niche expansion of ‘urban adapters’^29^ (sensu Blair 1996), and while this is most clearly observed in smaller omnivores who directly exploit human waste and hunt synanthropic prey (e.g., coyotes^30, 31^), this trend has also been reported in obligate carnivores. For example, SIA reveals that niche expansion in puma (*Puma concolor*) persisting on the fringe of a rapidly developing North American city has followed human expansion over space and time, with a significant shift to consumption of exotic and invasive species^15^. With increasing development pumas may also shift their diet to predate on a greater diversity of prey, and a higher proportion of smaller prey^32^. Additionally, human disturbance is linked to community-level shifts in predators, with increased trophic niche overlap (e.g., coyotes, bobcats, and grey foxes^33^; seven sympatric apex and mesocarnivores^34^). The net effect of changes in the prey base of urban-adapted predators is complex, but can be successfully revealed with stable isotopic signatures^35^. For example, a recent global meta-analysis of multiple predator taxa revealed that δ^13^C isotopic ratios were consistently more enriched in cities, suggesting a global pattern of increased consumption of human subsidies^27^. Changes in trophic ecology with increasing human transformation as inferred from δ^15^N isotopic ratios are, however, less clear. While urbanisation can reduce top-down control through multiple mechanisms, it can also increase bottom-up forcing through greater availability of anthropogenic foods^36^. In this way, predation rates in human landscapes can either be amplified by increased prey densities, or relaxed because of an abundance of easily accessible anthropogenic subsidies^27^ and, in so doing, create an urban ‘predation paradox’^3, 36, 37^. Evidence suggests that a predation paradox may be occurring in the cities of the Global North, although more research is required in different climatic zones across the world to determine to what extent predation relaxation and predator proliferation are characteristic features of landscapes undergoing urbanisation^36^.

In this study we use SIA to explore changes in the trophic ecology of caracals (*Caracal caracal*) across human-impacted landscapes within and near the city of Cape Town, South Africa. Caracals are medium-sized (6–20 kg) and trophically mid-ranked predators across most of their range in Africa and the Middle East. With the historic extirpation of local populations of Cape leopards (*Panthera pardus pardus*) and lions (*P. leo melanochaita*) in the Cape Town area, caracals have become the de facto apex predator^38, 39^, although leopards do persist in low densities in mountainous areas north and east of the city sprawl^40^. As highly adaptable generalists, caracals exploit a wide array of prey resources, but generally focus on the most abundant^41, 42^. Around the metropole of Cape Town individuals alter their foraging behaviour with increasing exposure to urbanisation, hunting closer to human development and likely taking advantage of abundant prey which benefit from human resource subsidies^39^. Feeding event data from scat and GPS kill-site clusters indicate that despite the presence of abundant exotic prey, such as introduced species and domestic animals, caracals focus on native, wild and synanthropic (i.e., human associated) prey^43^. The importance of human subsidies and niche shifts in this peri-urban caracal population, however, remain difficult to quantify, as feeding event detection by both scats and GPS clusters has methodological biases^43^. To better understand the influence of rapid environmental change on the trophic ecology of urban adaptors like caracals, we analysed stable isotope ratios within a model-based approach to test the relationship between increasingly transformed human landscapes and trophic niche flexibility in our study populations. To do this we first assess potential ﻿modification of δ^15^N and δ^13^C values through enrichment in urban and agricultural systems as measured by the Human Footprint Index^44^, by determining (i) the δ^15^N-based trophic level of both urban and rural caracal populations to understand how foraging near urban areas influences trophic position, and (ii) the relative importance of human-subsidised food resources by examining δ^13^C patterns in individuals using rural and urban areas. We then determine and compare the isotopic niche width and overlap between caracals in both urban and rural landscapes across the study area, and between demographic groups (age and sex) in these areas to test for niche expansion in human-transformed landscapes.

## Methods

### Study sites

**Figure 1:**
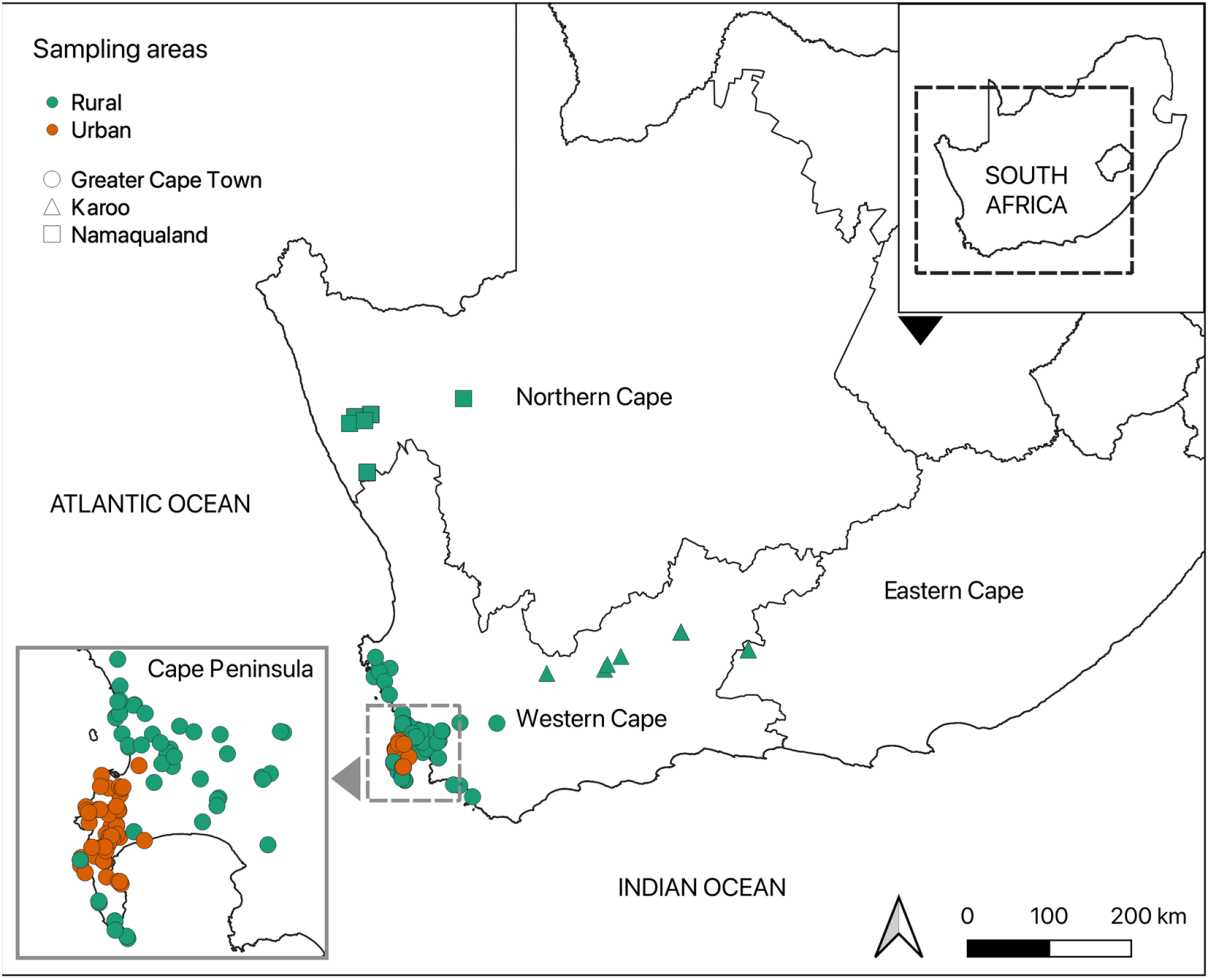
Map of caracal sampling locations in rural (green) and urban (orange) areas as determined by the Human Footprint Index across the Greater Cape Town area (circles), Karoo (triangles) and Namaqualand (squares).

We compared caracal foraging ecology across an urban gradient, including individuals living in and around Cape Town, as well as individuals in more rural areas of the Karoo and Namaqualand regions of the Western Cape and Northern Cape of South Africa (Fig. 1). Samples were collected from around Cape Town, including the Cape Peninsula and Greater Cape Town area. The Cape Peninsula has a strong north–south urban gradient mainly due to the Table Mountain National Park dominating in the south, and is isolated from the rest of the country by the extensive urban sprawl of Cape Town’s metropole. Natural areas are characterised by highly biodiverse fynbos vegetation which grows on heavily leached sandstone soils with few nutrients^45^. The mean annual rainfall in the city is 600–800 mm/y^45^. The Greater Cape Town metropole area is a mosaic of residential, industrial, and agricultural land (mainly vineyards, cropland, and orchards). Samples from the Karoo were mainly from the Lainsburg region (Western Cape), in the semi-arid Great Karoo, and the Rietbron area (Eastern Cape), both of which are rural areas characterised by livestock agriculture (mainly sheep farming). This area falls into the Nama Karoo, with higher nutrient shale soil. The mean rainfall in this southern section of the Nama Karoo is around 500 mm/y^46^. The samples from Namaqualand were from Kamiesberg, Northern Cape. This area is classified as Lowland Succulent Karoo, which is associated with varied geology, including base-rich shallow sands and sandy loams, and overlying bedrock (mostly granite gneiss but also quartzite) and hardpans (calcrete and ‘dorbank’, a reddish-coloured hardpan cemented by silica), and quartz fields^47^. The Kamiesberg region receives a rainfall of around ﻿400 mm/y^47^.

### Sample collection

Fur samples from the shoulder were collected from caracals while anaesthetised during GPS collar deployment (n = 26 samples) and from opportunistically collected mortalities (n = 137), mainly comprising carcasses retrieved from roadkill (urban areas) and lethal predator control (rural areas). ﻿Full GPS collar deployment and sample collection details are reported in Serieys et al.^48^ and Leighton et al.^43^. For the Cape Peninsula area, animal capture, handling, and sampling protocols followed ethical guidelines approved by the American Society of Mammologists and the University of Cape Town Animal Science Faculty Animal Ethics Committee (2014/V20/LS) in accordance with ARRIVE guidelines^49^, and samples were collected under permit from Cape Nature (AAA007-0147- 0056), and South African National Parks (SERL/AGR/017-2014/V1). Caracals were sampled from the Karoo with a CapeNature permit (no. 0056-AAA007-00161), and from Namaqualand with permission from University of Cape Town Science Faculty Animal Ethics Committee (2013/V30/BC), South African National Parks (CRC-2013/029-2014) and Northern Cape Department of Environment and Nature Conservation (FAUNA 1157/2013; FAUNA 1158/2013).

### Sample preparation for ^15^N/^14^N and ^13^C/^12^C isotope analysis

Fur samples were used to determine individual isotopic ratios. Fur samples likely represent a few months of diet^50^, although there is no data on caracal fur growth rates or isotope turnover rates. The fur sub-samples were obtained from existing samples with at least 1 mg weighed out and placed in Eppendorf tubes. Samples were degreased using a cleaning solution of chloroform, methanol and distilled water in a ratio of 2:1:0.8 as described in Bligh and Dyer^51^ and following Lee-Thorp et al.^52^. The fur samples were immersed in the degreasing solution, shaken, and left for at least 24 h. The degreasing solution was then removed using a pipette and the fur was then washed with 1 ml distilled water three times and shaken before being drained. The fur samples were then left in the open tubes and dried at 40 °C for at least 24 h in a drying oven.

Cleaned samples were processed in the Archaeology Department’s Isotope Laboratory at the University of Cape Town or the Stable Isotope Laboratory in the Mammal Research Unit of the University of Pretoria. The fur samples were weighed (0.3–0.5 mg) with a micro balance (Sartorius M2P micro balance) before being placed into tin cups to 1 mcg accuracy and folded for final processing. These were then combusted in a Delta V Plus organic elemental analyser/isotope ratio mass spectrometer (IRMS) via a Conflo IV gas control unit (Thermo Scientific, Germany). The calibrated standards used by both laboratories were DL Valine, Merck Gel, sucrose and Choc, which are calibrated against International Atomic Energy Agency (IAEA) standards. Nitrogen is expressed relative to atmospheric nitrogen, carbon is expressed relative to Pee-Dee belemnite^24^.

### Spatial variables

For each mortality individual, we calculated a buffer polygon which was sized according to mean home range sizes for adult male, juvenile male and female caracals (see Leighton et al.^53^ for details). For collared individuals we calculated 95% local convex hull (LoCoH) home range estimates using the *T-LoCoH* package in R (see Leighton et al.^53^ for more details). We calculated the mean Human Footprint Index (HFI, a score 0–50) 2018 release^44, 54, 55^ within each home range or buffer polygon. HFI is an effective proxy for human development and disturbance as it incorporates built environments, human population density, electric infrastructure, crop lands, pasture, roads, railways and navigable waterways into a single metric at a global scale^44^. Home ranges or buffers with < 25 HFI were classified as ‘rural’, while all other locations (≥ 25; i.e., half the HFI score) were classified as ‘urban’. This split was reasonable based on classification of known individuals. According to this classification, rural areas are those outside of a town or city, with low population density and small settlements with or without agricultural activity. This resulted in 92 urban and 76 rural individuals. The strong aridity gradient in South Africa is known to affect isotope ratios, where lower rainfall is strongly associated with higher δ^15^N^56–58^. An aridity index raster was therefore calculated by dividing precipitation with evapotranspiration using high-resolution (< 5 km^2^) data from TerraClimate (climatologylab.org/terraclimate.html)^59^.

### Statistical analysis

Differences in δ^15^N and δ^13^C values in caracal fur between areas (urban vs. rural; Fig. 1), sex (male vs. female) and age classes (adult vs. juvenile) were tested using MANOVAs followed by post-hoc one-way ANOVAs and Tukey tests. Multivariate ellipse-based metrics calculated in SIBER (Stable Isotope Bayesian Ellipses in R; Jackson et al.^60^) were used to test the isotopic niche width of the caracal groups and to determine levels of niche overlap. Bayesian ellipses calculate isotopic niche as Standard Ellipse Area (SEA), and SEA corrected for small sample size (SEAc), and niche overlap as the proportional ellipse overlap between groups. Overlap is calculated as the percentage of the non-overlapping area of the two ellipses of specified groups. We then calculated the proportions, and hence probability, that the posterior ellipse areas differed for sex and age classes between sites (i.e., rural vs urban). To understand how isotope signatures changed with increasing human development while accounting for aridity, we ran linear models (using *lm*) for δ^13^C and δ^15^N as dependent variables with area (categorical variable: urban vs rural), HFI, and aridity index as independent variables. ﻿We selected the top model using AICc^61^ by fitting models with all covariate permutations using the *MuMin* package^62^. ﻿To test whether potential patterns were maintained at a local scale, we also ran models with only the individuals in the Greater Cape Town area (i.e., excluding Karoo and Namaqualand samples; n = 128). Collinearity between variables was assessed with variance inflation factors (VIF < 2). All statistical analyses were conducted using R version 4.0.2^63^ (2020-06-22). Plots were created in R using *SIBER* and *ggplot2* and the map was created using QGIS version 3.28.6^64^.

## Results

Caracal fur sampled across the study area showed a wide range of both δ^13^C (mean = -20.53 ± 1.80, minimum = -24.37, maximum = -15.22 ‰) and δ^15^N values (mean = 10.60 ± 3.20, minimum = 5.37, maximum = 21.78 ‰).

### Caracal isotopic ecology in urban and rural areas

Just over half of the samples (55%) were analysed from individuals originating from urban areas (HFI > 25; Table 1).

**Table 1:** Mean ± SD and variance of δ^13^C and δ^15^N values of caracal fur samples analysed from both urban and rural areas of South Africa as determined by Human Footprint Index.

Site	n	δ^13^C	δ^13^C variance	δ^15^N	δ^15^N variance	Human Footprint Index
Rural	76	− 20.86 ± 1.86^a^	3.47	12.26 ± 3.68^a^	13.54	11.30 ± 6.43
Urban	92	− 20.26 ± 1.70^b^	2.09	9.23 ± 1.86^b^	3.45	32. 80 ± 4.10

Within columns, different superscript letters indicate significant differences (*P* < 0.05) between paired values, estimated from Tukey post-hoc tests. Mean ± SD human footprint index values are reported for each site.

**Figure 2:**
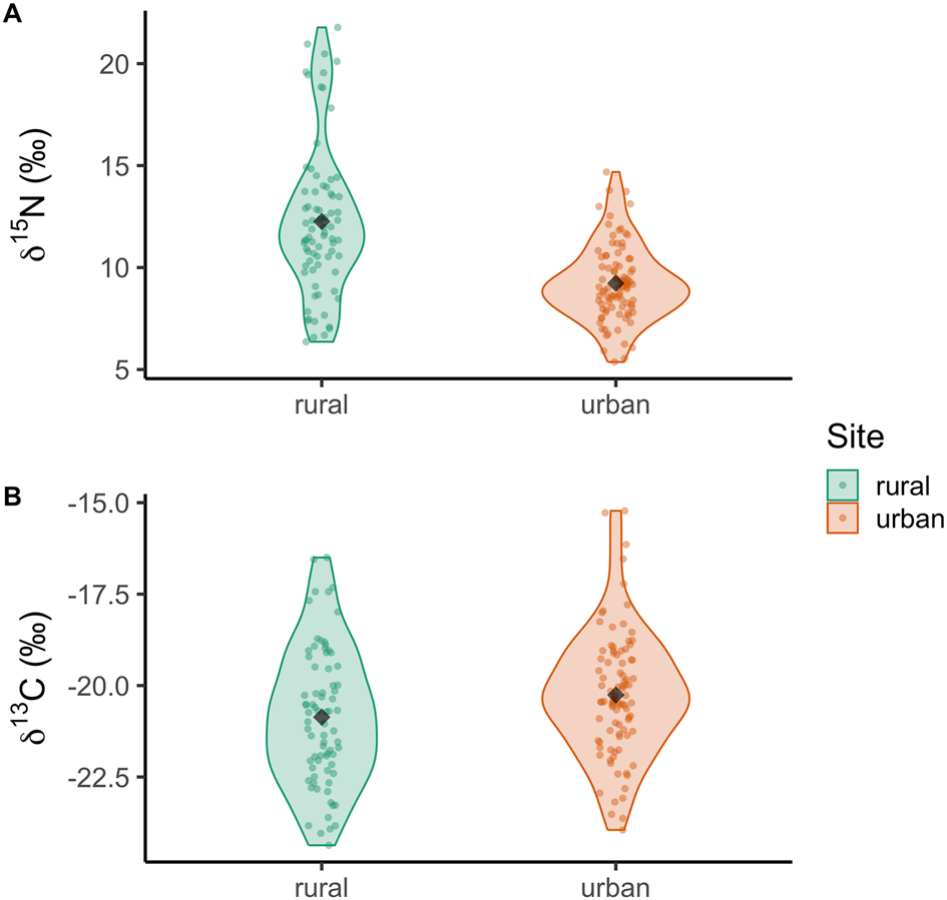
Violin plots with raw data points, and means plotted as diamonds for (**A**) δ^15^N (F_1,161_ = 47.68, *P* < 0.001) and (**B**) δ^13^C (F_1,161_ = 4.82, *P* < 0.05) in caracal fur from urban (n = 92) and rural (n = 76) areas of the South African study region.

Overall, site (rural vs urban) was significantly associated with both δ^13^C and δ^15^N (MANOVA; Pillai’s Trace test statistic = 0.31, *P* < 0.001; large effect size, ηp^2^ = 0.31). The ANOVAs showed that urban and rural caracals had significantly different δ^15^N values (*F*_1,161_ = 47.68, *P* < 0.001), where Tukey post-hoc tests revealed caracals from rural areas had a relatively more enriched δ^15^N value range compared to the urban group (Table 1, Fig. 2). Further, the urban and rural caracals had significantly different δ^13^C values (*F*_1,161_ = 4.82, *P* < 0.05), where Tukey tests revealed that caracals sampled from urban areas were more enriched in δ^13^C than those sampled in rural areas (Table 1, Fig. 2). Additionally, there was decreased variance in both δ^13^C and δ^15^N values with increased urbanisation (Table 1).

### Spatial analysis of δ^15^N and δ^13^C signatures

According to model selection based on AICc, the top linear model for δ^15^N included an interaction between sampling site (i.e., urban vs rural) and aridity (see Table S1 for full model results).

**Figure 3:**
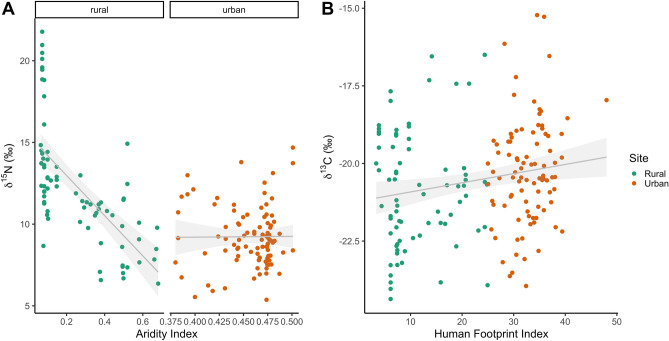
Relationship between (**A**) aridity δ^15^N (F_1,165_ = 81.53, P < 0.01) and (**B**) Human Footprint Index (F_1,161_ = 6.5821, *P* < 0.05) δ^13^C values for caracals sampled in urban and rural areas. Shaded areas represent 95% confidence intervals.

**Figure 4:**
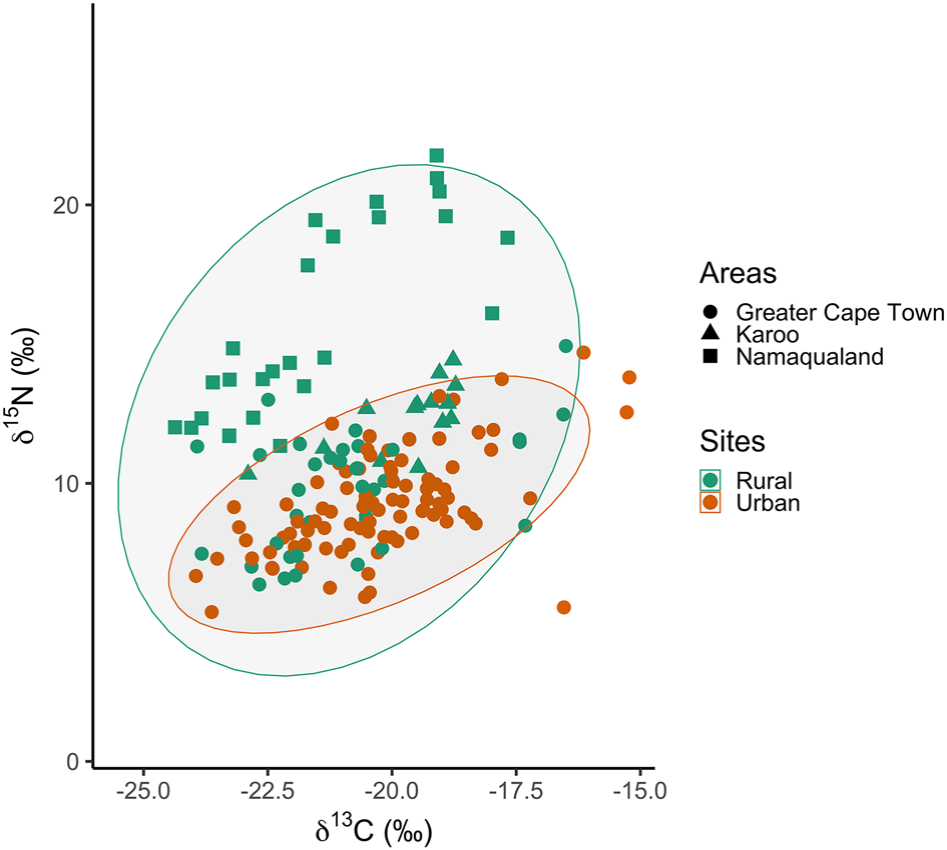
Isotopic niche space of caracal fur sampled in urban (n = 92) and rural (n = 76) areas of the South African study region. Higher δ^15^N indicates feeding on nitrogen enriched prey, while higher δ^13^C indicates feeding on anthropogenic food subsidies through prey consumption of C_4_ plants.

Overall, as mean aridity in home ranges or buffers increases, caracal fur is less enriched with δ^15^N (*F*_1,165_ = 81.53, *P* < 0.01; Fig. 3), an effect that was more pronounced in rural areas where the range of aridity values was higher. The effect of aridity was also maintained on the local scale for the Greater Cape Town area (i.e., excluding Karoo and Namaqualand individuals; *F*_1,126_ = 6.69, *P* < 0.05). This aridity effect is particularly driven by rural individuals in the Namaqualand region of the country (see Fig. 4). Importantly, this trend was also significantly influenced by sampling site, where urban individuals (mean = 9.23 ± 1.86 ‰) had lower δ^15^N than rural (mean = 12.26 ± 3.70 ‰; Table 1) individuals overall (site: *F*_1,164_ = 70.8166, *P* < 0.01; Fig. 3). In contrast, model selection based on AICc revealed that the top model for δ^13^C included only a positive association with HFI, and not site. As human footprint increases, caracal fur showed significantly more enriched δ^13^C signals (HFI: *F*_1,161_ = 6.5821, *P* < 0.05; Fig. 3). The site (urban vs rural) was not included in the top model, suggesting the continuous HFI was a better explanatory variable than this categorical variable. This trend was conserved when only caracals around Cape Town were considered (i.e., excluding Karoo and Namaqualand individuals; *F*_1,126_ = 6.7819, *P* < 0.05). The R^2^ values for these models were low (Table S2), likely due to high interindividual and interpopulation variation, and suggesting that the addition of other spatial explanatory variables may explain more of the variation in δ^13^C values.

### Isotopic niche space for urban and rural caracals

To compare isotopic niche between caracals in urban and rural areas we fitted Bayesian models and calculated their associated ellipse parameters (Fig. 4).

The posterior distribution of Bayesian ellipses revealed that urban caracals had a smaller overall isotopic niche than rural caracals (100% of posterior ellipses were larger for rural caracals; Table 2, Fig. 4 and Fig. S1). There was a relatively low overlap of 36.55% of total ellipse area for urban and rural caracals (Fig. 4). Although overlap was higher (63.75%), this pattern of smaller isotopic niche in urban caracals was maintained when considering only individuals around Greater Cape Town (i.e., excluding individuals from arid areas of Karoo and Namaqualand; 91.53% of posterior ellipses were larger for rural caracals).

**Table 2:** Bayesian ellipse parameters for fur samples of caracals from rural and urban areas across the study region, including for subsets of individuals for which demographic information was available.

	Urban					Rural				
	All	Male	Female	Juvenile	Adult	All	Male	Female	Juvenile	Adult
*n*	96	49	39	54	37	72	19	16	18	54
TA	55.34	33.94	18.13	47.01	20.93	82.00	27.74	23.10	40.60	74.07
SEA	8.11	8.25	4.66	9.85	4.88	20.61	9.63	9.82	11.71	22.07
SEAc	8.20	8.42	4.80	10.04	5.02	20.89	10.23	10.52	12.44	22.50

The TA (total convex hull area), SEA (standard ellipse area) and SEAc (SEA corrected for small sample size) are based on the maximum likelihood estimates of the means and covariance matrices of each group.

### Isotopic niche patterns by sex and age

Demographic information on sex and age was available for a subset of individuals (sex: n = 123, 68 male and 55 female; age: n = 163, 91 adults and 72 juveniles, including subadults and kittens) and these were used to explore niche width and overlap across these demographic parameters in rural and urban individuals.

A MANOVA showed that site (rural vs urban; Pillai’s Trace test statistic = 0.06, *P* < 0.05; medium effect size, ηp^2^ = 0.06) and sex (male vs female; Pillai’s Trace test statistic = 0.05, *P* < 0.05; medium effect size, ηp^2^ = 0.05) were significantly associated with both δ^13^C and δ^15^N values (Table 3).The ANOVAs with post-hoc Tukey tests further revealed that this association was not significant for δ^15^N for either site or sex (*P* > 0.05). However, males had enriched δ^13^C values (*F*_1,120_ = 5.49, *P* < 0.05; Table 3, Fig. S2) regardless of site (*F*_1,120_ = 1.79, *P* = 0.18; Fig. S2). There was relatively high overlap between males and females in both the urban (mean overlap area of 64.34% of the total ellipse area) and rural areas (56.33% of the total ellipse area), indicating less distinction between the groups in terms of isotopic niche space (Fig. S3). Nevertheless, the Bayesian ellipses revealed that male isotopic niche was generally larger than females in both rural (44.38% of the male posterior ellipses were larger than females) and urban areas (99.3% of male posterior ellipses were larger; Fig. S3 and S4). Further, rural caracals had a larger isotopic niche than those in urban areas, for both males (68.9% of posterior ellipses were larger for rural males) and females (99.28% of posterior ellipses were larger for rural females; Fig. S3 and S4).

**Table 3:** Mean ± SD, minimum and maximum of δ^13^C and δ^15^N values of fur samples of caracals of differing demographics from rural and urban areas of the South African study region.

Site	Demographic	n	Mean δ^13^C	Min δ^13^C	Max δ^13^C	Mean δ^15^N	Min δ^15^N	Max δ^15^N
Rural	Female	16	− 20.80 ± 1.78	− 23.92	− 16.55	9.97 ± 1.94	6.36	12.46
	Male	19	− 20.74 ± 1.91	− 23.83	− 16.50	9.34 ± 2.07	6.58	14.92
	Juvenile	18	− 20.98 ± 1.82	− 23.92	− 16.50	9.66 ± 2.19	6.58	14.92
	Adult	54	− 20.76 ± 1.93	− 24.37	− 16.55	13.20 ± 3.78	6.36	21.78
Urban	Female	39	− 20.85 ± 1.27	− 23.63	− 17.96	8.88 ± 1.51	5.37	11.58
	Male	49	− 19.87 ± 1.86	− 23.95	− 15.22	9.56 ± 2.00	5.91	14.69
	Juvenile	54	− 20.11 ± 1.85	− 23.95	− 15.22	9.42 ± 2.14	5.54	14.69
	Adult	37	− 20.45 ± 1.48	− 23.63	− 17.22	8.87 ± 1.26	5.37	11.68

For age class, the MANOVA revealed that both site (urban vs. rural; Pillai’s Trace test statistic = 0.32, *P* < 0.001; medium effect size, ηp^2^ = 0.32) and age (adult vs. juvenile; Pillai’s Trace test statistic = 0.04, *P* < 0.05; small effect size, ηp^2^ = 0.04) was significantly associated with both δ^13^C and δ^15^N values. The ANOVAs further revealed significant differences between groups for δ^15^N values (Table 3), where Tukey tests showed that rural caracals had higher values than urban caracals (*F*_1,160_ = 34, *P* < 0.001; Fig. S5), and adults were more δ^15^N enriched than the juveniles (*F*_1,160_ = 19.38, *P* < 0.001). Tukey tests indicated that urban caracals had marginally significantly higher δ^13^C values than rural caracals (*F*_1,160_ = 3, *P* = 0.09; Fig. S5) regardless of age (*P* > 0.05). The Bayesian ellipse analysis revealed relatively high overlap levels between juveniles and adults in both the urban (mean overlap area of 47.13% of the total ellipse area) and in rural areas (49.51% of the total ellipse area; Fig. S6; Table 2). However, analysis of the Bayesian ellipses also revealed that in urban areas juveniles had a greater isotopic niche than adults (99.95% of juvenile posterior ellipses were larger than adults) but not so for rural areas, where adult niche was larger (98.32% of adult posterior ellipses were larger; Fig. S7; Table 2). Further, caracals in urban areas had smaller isotopic niches, for both adults (100% of the posterior ellipses were larger for rural adults; Fig. S7) and juveniles (75% of the posterior ellipses were larger for rural juveniles; Fig. S7).

## Discussion

We used SIA to explore the foraging ecology of an adaptable carnivore across a gradient of urbanisation and human impact in the Cape region of South Africa. Our results reveal the extent to which rapid environmental change is altering the ecological role of predators, specifically within an urbanising system. Across our study area, caracals show a wide range of δ^13^C and δ^15^N values, indicating remarkable dietary flexibility. However, adaptation to urbanisation significantly influences their isotopic niche width and trophic dynamics, with marked differences between urban and rural communities and demographic groups.

### Urban niche contraction in an adaptable carnivore

Overall, caracals living in more urban areas have a narrower isotopic niche and were particularly depleted in δ^15^N compared to those individuals found in more rural areas. This was unexpected given literature suggesting expansion of wildlife niches in response to human disturbance and changing prey availability^65–67^ *vis a vis* the Niche Variation Hypothesis. This hypothesis posits that niche expansions occur at a population level with increased inter-individual variation in resource use primarily due to release from interspecific competition^68–70^. This literature is largely supported by studies on biological invasions, where populations released from interspecific competition are observed to become more generalised^71–74^. Further, the results reported here are in contrast to, for example, previous work on an apex predator, the puma, suggesting niche expansion due to diet shifts in urban areas to incorporate novel prey^15^. However, our results are in agreement with a more recent study on an iconic mesopredator urban adaptor, the red fox (*Vulpes vulpes*), which found narrower isotopic niches and lower δ^15^N values in urban individuals^75^.

This pattern of niche contraction in urban areas is also retained when accounting for sex and age in caracals. A detailed analysis of those individuals with demographic information revealed that juvenile caracals had broader niches than adults in more urbanised areas, likely due to both the use of more varied landscapes while still establishing their home range and consuming a wider variety of small prey species (i.e., they exhibit less specialised diets than adults^43^). This is also supported by analysis of the habitat selection of Cape Peninsula caracals revealing larger home ranges in more urbanised areas^39^, and by scat and GPS cluster analysis revealing juveniles feeding more evenly across prey groups than adults^43^. On the Cape Peninsula region of our study, where most ‘urban’ classified individuals originated, diet analysis reveals that adult caracals feed on many prey species (> 65 species); but in terms of frequency of occurrence and biomass consumed, they focus on only a few species^43^, including Guinea fowl (*Numida meleagris*), Egyptian geese (*Alopochen aegyptiaca*), vlei rats (*Otomys irroratus*), and antelope, all of whom are primary consumers. Adult male caracals had broader isotope niches than females, likely reflecting their larger home ranges and body size^39^, as well as feeding on a more diverse prey base than the smaller females (males: 57 species, females: 48 species^43^). This hyperfocus on a few low trophic level species likely explains the narrower isotopic niche in the urban caracal community. Importantly, changes in trophic level and trophic niche of carnivores, such as increases or decreases in the level of carnivory or specialisation, have cascading effects with whole ecosystem impacts^14, 36, 76^.

### Dietary and trophic shifts with increasing human footprint

Spatial analysis of isotopic signatures revealed that increasing anthropogenic pressure (i.e., higher HFI) was associated with enriched δ^13^C values but that δ^15^N values were lower in urban areas, even when accounting for aridity. Although variation in δ^13^C was high for caracals in the study area, likely due to interindividual and site differences, this trend was significant. Similar increases in δ^13^C have been reported for other carnivores using human-transformed areas (e.g., red foxes^75^, kit foxes^77^, coyotes^30^, and black bears^78^), suggesting a high input of anthropogenic food sources (e.g., δ^13^C enriched C_4_ photosynthetic plants) being directly consumed or consumed by prey. In our study system it is likely that caracals in and around urban Cape Town incorporate more synanthropic (i.e., human associated native species) and exotic (i.e., domestic and introduced species) prey into their diet who benefit from human subsidies. This is similar to SIA of urban coyotes who have been found to ﻿consume more protein-poor food than their rural counterparts, as a result of anthropogenic subsidies^30^. Together, our results on a carnivore in the Global South corroborate the findings of a recent meta-analysis^27^, which demonstrates that urban predators have variable trends in δ^15^N but are usually enriched in δ^13^C. The meta-analysis concluded that consumption of enriched carbon food items increased in cities due to direct and indirect consumption by prey species of corn, wheat and sugar-rich anthropogenic refuse^27^. In general, urban wildlife may shift their preference to anthropogenic food over natural sources because of constant availability, predictability, and lower foraging costs^16^, particularly for omnivores, like red foxes^79^, gulls^80^ (*Larus hyperboreus*) and dingoes^14^ (*Canis lupus dingo*). However, obligate carnivores like pumas^15, 32^, bobcats^81, 82^ and caracals also clearly shift their diets to exploit the availability of synanthropic prey, and thereby benefiting from a stable supply of anthropogenic foods, albeit indirectly.

The higher δ^15^N values in those caracals from rural areas, particularly from Namaqualand, may indicate higher trophic level feeding (i.e., not majority primary consumers), which represents greater incidence of feeding on other carnivores (i.e., secondary or tertiary consumers). On the Cape Peninsula, carnivore species comprise ~ 5% of caracal diet (e.g., large spotted genet, *Genetta tigrina,* and Cape grey mongoose, *Galerella pulverulenta*^43^). In more rural areas, this proportion has been reported as higher. For example, in the Karoo, Stuart^83^ reported 5.2% occurrence of carnivores in caracal diet, while Drouilly et al.^84^ report 7.8%. In agricultural areas of the southern Cape, occurrence of carnivores in caracal diet ranges from ~ 5–12%^85^. However, there are also studies reporting lower occurrence in caracal diet, including in Karoo National Park^86^ (2%), Namaqua National Park^87^ (< 2%), and the Bedford district of the Eastern Cape^88^. Further, caracals in the rural Karoo and Namaqualand commonly feed on sheep^84, 87, 88^, which have significantly elevated δ^15^N in these areas (~ 10‰) compared to the southern Western Cape (~ 5‰)^89, 90^. It should be noted that scat and GPS cluster feeding sites provide only a snapshot of diet, limiting their comparative value to the integrated signatures of SIA. Importantly, in South Africa, plant and animal δ^15^N values vary strongly with rainfall^56–58^, with high δ^15^N values (> 10 ‰) for herbivores occurring in areas receiving less than 400 mm of rain per annum^91^, as in the Karoo and Namaqualand. Our results also reveal a strong positive association between δ^15^N and aridity, particularly in the Namaqualand individuals. This trend is likely due to the adaptive ﻿increased excretion of isotopically light urea, which allows prey animals in these arid areas to reduce their volume of urinary output^91^. Further, the open nutrient cycling in ﻿water-limited systems in southern Africa means the resulting natural abundance of plant foliar ^15^N in these systems is enriched^56^. The higher δ^15^N values of caracal fur in the rural areas may also reflect the nutrient-enriched soils across the distinct geologies of the Namaqualand and Karoo rural sites compared to the Cape Town area^20^, which is known for nutrient-poor soils^45, 92^.

### Stable isotope insight into the urban ‘predation paradox’

Increasing evidence suggests that through providing augmented resource subsidies, anthropogenic systems may decouple predator–prey relationships^3^, thereby creating an urban ‘predation paradox’^36^. The predation paradox is created when reductions in predation rates (i.e., predation relaxation) and increases in predator abundance (i.e., predator proliferation) lead to conflicting predictions on the effects of top-down control in urban and non-urban environments^36^. ﻿The predation rate of a given population is represented by the proportion of that population killed by predators per unit time. In urban areas predation pressure may decrease because a smaller proportion of a given population succumbs to predation than in less developed areas^93^. According to the predation paradox hypothesis, low predation rates can be a result of predator subsidy consumption, prey hyperabundance, and prey specialisation^36^. For example, a recent meta-analysis found that predation rates on birds were significantly higher in rural than urban habitats, signifying reduced predation pressure with increasing urbanisation^37^.

Our results suggest that an urban predation paradox may characterise the rapidly developing area of Cape Town and its surrounds. Here, focused predator subsidy consumption may occur because of predictable, anthropogenic subsidies to synanthropic prey; because these are predominantly primary consumers, the resulting shifts in dietary composition of caracals towards lower trophic level prey, results in reduced δ^15^N values and increased δ^13^C values. Anthropogenic subsidies certainly lead to hyperabundance of prey in the urban areas of our study, such as Guinea fowl and Egyptian geese, which are found in much higher densities closer to Cape Town’s urban edge^94^ and are likely buffered from the effects of top-down regulation. As a result, caracals using their preadapted ambush hunting technique have likely specialised on these species because of their relative abundance around urban areas, thus reducing predation pressure on alternative prey species. A better understanding of the predation paradox and the role of anthropogenic subsidies in predator proliferation in our study area requires detailed data on caracal abundance in and around Cape Town. Importantly, given their highly opportunistic diet, an increase in the caracal population in and around urban areas driven by synanthropic prey consumption could negatively impact wild, indigenous species that are not buffered by human subsidies^3, 95^, including rare or endemic birds and reptiles. For example, several threatened species, such as the Black oystercatcher (*Haematopus moquini;* Near Threatened), African penguin (*Spheniscus demersus*; Endangered), Cape cormorant (*Phalacrocorax capensis*; Endangered), and Bank cormorant (*Phalacrocorax neglectus*; Endangered), occur in the diet of Cape Peninsula caracals^43^. Unfortunately, while caracals appear to be breeding successfully in our study area, there is no associated temporal data on population trends.

In summary, our findings point to trophic shifts in an adaptable obligate carnivore driven primarily by anthropogenic subsidies in a rapidly urbanising region of the Global South. SIA results suggest that urban caracals have shifted to lower trophic level prey compared to rural caracals. Under a scenario where urban top predators focus mainly on low trophic level prey, their functional role in top-down processes is likely to be substantially altered relative to their rural counterparts. Here, we argue that a trend of narrowing isotopic niche and lower trophic level feeding points to ‘trophic downgrading’ of urban-adapted caracal in our study system (sensu Estes et al.^96^). Rather than ascending to the position of apex predators (e.g., in Cape Town via the local extinction of leopard and lion), the altered ecology of mesopredators in human-transformed landscapes can result in dampening of their top-down control^10^. Further research is needed to determine the degree to which this pattern characterises other adaptable carnivores in human-transformed landscapes. Ordiz et al.^11^ argue that humans have demoted large carnivores from their apex position in food webs but do not substitute their ecological roles. The removal of apex predators in human landscapes with concurrent provision of resources, may ironically limit the ecological influence of medium-sized carnivores as ecosystem regulators, and likely points to a widespread disruption of trophic dynamics in the world’s rapidly urbanising areas.

### Supplementary Information

Supplementary Information.

## Data Availability

The datasets used and analysed during the current study available from the corresponding author on reasonable request.
