# Supplementary material for: Trophic downgrading of an adaptable carnivore in an urbanising landscape
Source: Sci Rep. 2023 Dec 7;13:21582. doi: 10.1038/s41598-023-48868-x (PMC10703923; doi:10.1038/s41598-023-48868-x)
Supplement: Supplementary file 1 — Supplementary Information. [file 41598_2023_48868_MOESM1_ESM.docx]

**Trophic downgrading of an adaptable carnivore in an urbanising landscape**

Gabriella R. M. Leighton^1,2^, William Froneman^1^, Laurel E. K. Serieys^2,3,4^ & Jacqueline M. Bishop^2^

*^1^SARChI Chair in Marine Ecology, Department of Zoology and Entomology, Rhodes University, PO Box 94, Grahamstown 6140, South Africa*

*^2^Institute for Communities and Wildlife in Africa***,** *Department of Biological Sciences, University of Cape Town, Rondebosch, Cape Town 7701, South Africa*

*^3^Panthera, ﻿8 W 40th St, New York, NY 10018, USA*

*^4^ Cape Leopard Trust, Cape Town, South Africa*

**Supplementary material**

**Table S1** Demographic groups and δ^15^N and δ^13^C values (mean and SD) for urban and rural caracals in the South African study region

| **Site** | **Age class** | **Sex** | **n** | **Mean δ^13^C** | **SD** | **Mean δ^15^N** | **SD** |
| --- | --- | --- | --- | --- | --- | --- | --- |
| rural | adult | female | 6 | -20.2 | 2.55 | 9.49 | 2.63 |
|  |  | male | 9 | -20.7 | 1.7 | 9.46 | 1.65 |
|  |  | NA | 39 | -20.9 | 1.92 | 14.6 | 3.3 |
|  | juvenile | female | 8 | -21.2 | 1.24 | 9.91 | 1.44 |
|  |  | male | 9 | -20.6 | 2.3 | 9.08 | 2.57 |
|  |  | NA | 1 | -22.5 | NA | 13 | NA |
|  | NA | female | 2 | -21.3 | 0.791 | 11.6 | 0.334 |
|  |  | male | 1 | -21.6 | NA | 10.7 | NA |
|  |  | NA | 1 | -22.7 | NA | 11 | NA |
| urban | adult | female | 18 | -20.9 | 1.35 | 8.85 | 1.24 |
|  |  | male | 19 | -20.1 | 1.5 | 8.87 | 1.31 |
|  |  | NA | 1 | -19 | NA | 9.27 | NA |
|  | juvenile | female | 21 | -20.8 | 1.22 | 8.9 | 1.74 |
|  |  | male | 30 | -19.7 | 2.07 | 10 | 2.25 |
|  |  | NA | 2 | -18.4 | 2.65 | 6.53 | 1.4 |
|  | NA | NA | 1 | -21.2 | NA | 12.1 | NA |

**Table S2** Estimates (SE) for top linear models based on AICc for δ^15^N and δ^13^C and spatial variables for caracal fur samples across rural and urban areas in the South African study region (**P* < 0.1; ***P* < 0.05; ****P* < 0.01)

| **Variable** | **δ^15^N** | | **δ^13^C** | |
| --- | --- | --- | --- | --- |
| Dataset | All | Greater Cape Town | All | Greater Cape Town |
| Site (Urban) | -6.43* (3.78) |  |  |  |
| Aridity Index | -12.30*** (1.34) | -6.15** (2.38) |  |  |
| Site (Urban)*Aridity Index | 12.78 (8.31) |  |  |  |
| Human Footprint Index |  |  | 0.030** (0.011) | 0.046** (0.018) |
| Constant | 15.350*** (0.434) | 12.180*** (1.091) | -21.210*** (0.297) | -21.720*** (0.517) |
| R^2^ | 0.487 | 0.043 | 0.039 | 0.051 |
| AICc | 764.8 | 529.4 | 672.4 | 506.6 |
| Akaike weight | 0.241 | 0.258 | 0.339 | 0.288 |
| n | 168 | 128 | 168 | 128 |


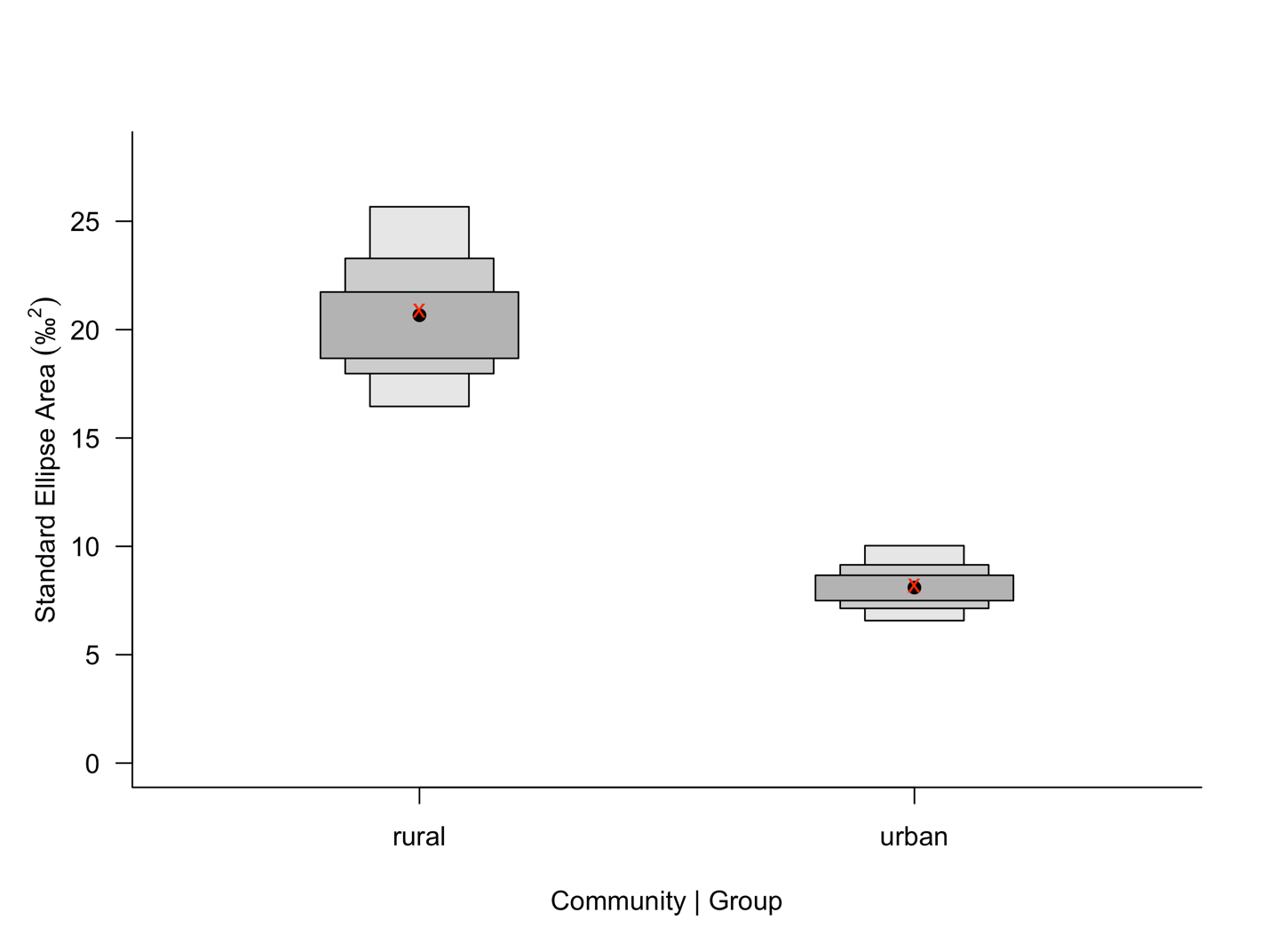


**Fig. S1** Density plot showing Bayesian standard ellipse area (SEA) estimates for each group (i.e., urban and rural). The red crosses indicate SEA estimates corrected for small sample size (SEAc)


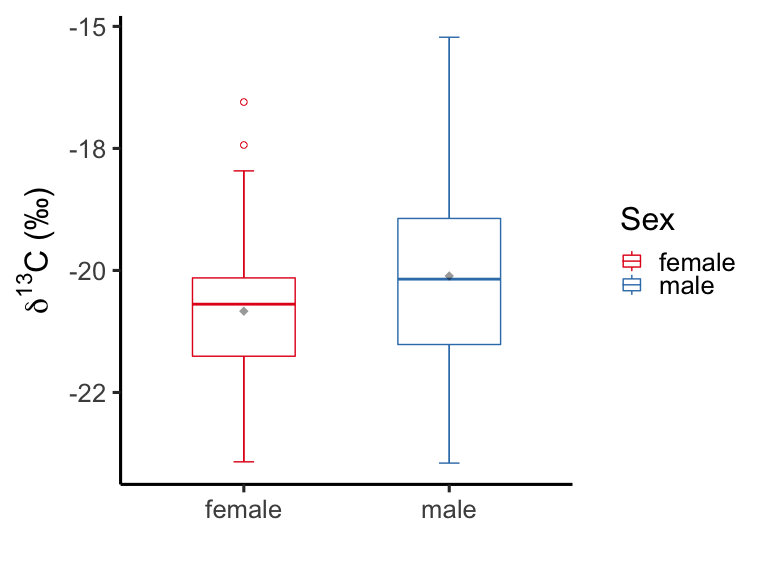


**Fig. S2** Boxplots with means plotted as diamonds for δ^13^C in female and male caracal fur from the South African study region


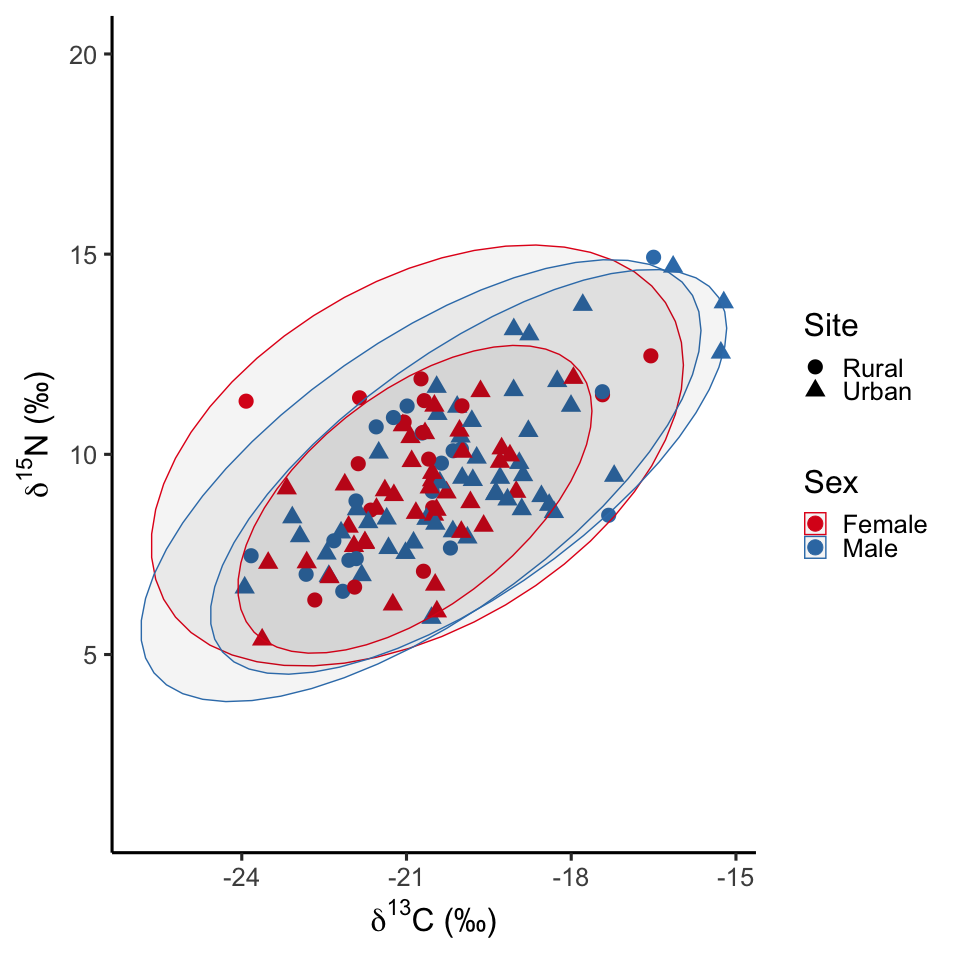


**Fig. S3** Isotopic niche of caracals separated by female and male individuals sampled in urban and rural areas


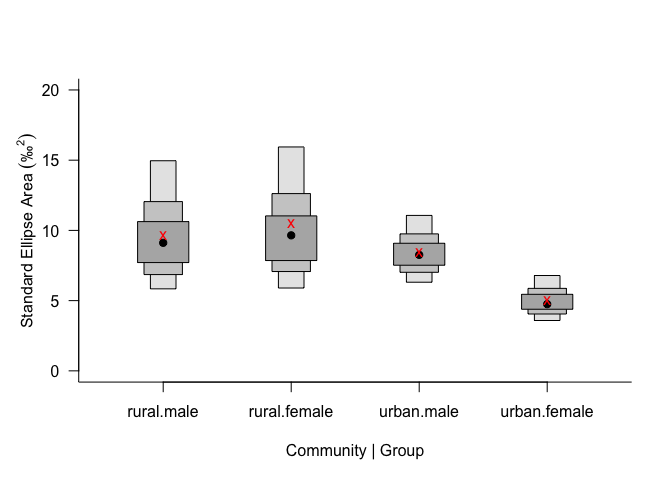


**Fig. S4** Density plot showing Bayesian standard ellipse area (SEA) estimates for each group (i.e., female and male) and community (i.e., urban and rural). The red crosses indicate SEA estimates corrected for small sample size (SEAc)


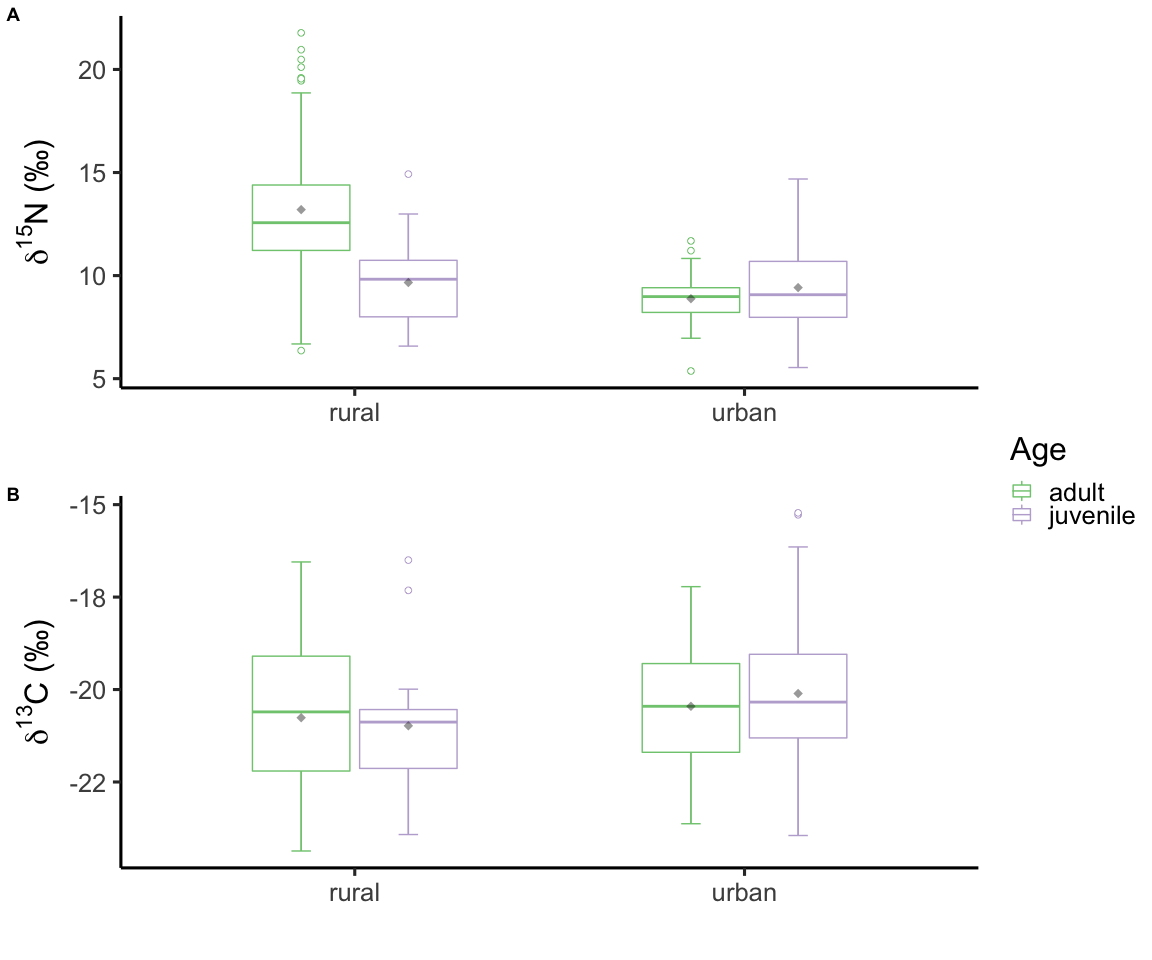


**Fig. S5** Boxplots showing 5-number summary with means plotted as diamonds for δ^13^C in female and male caracal fur from the South African study region


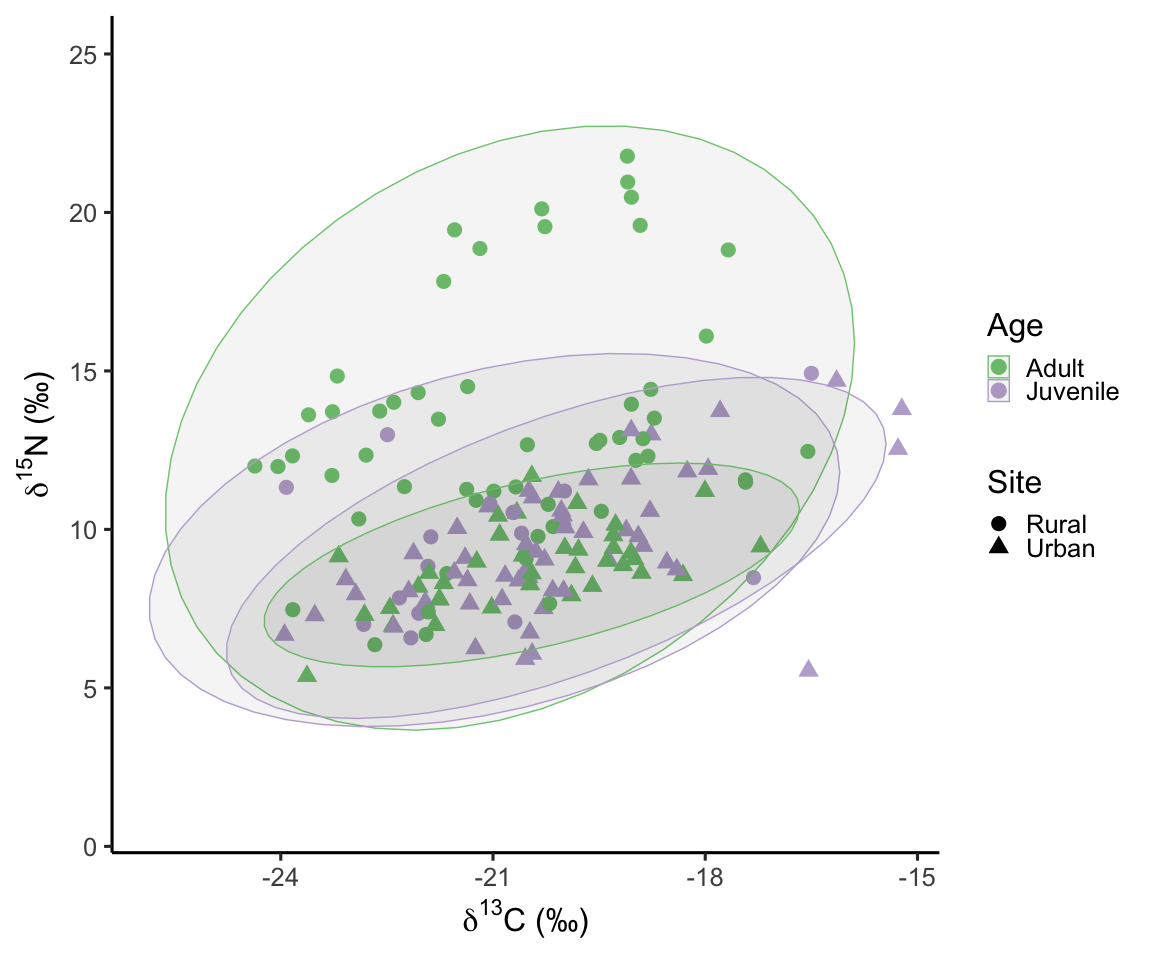


**Fig. S6** Isotopic niche of caracals separated by adult and juvenile individuals sampled in urban and rural areas


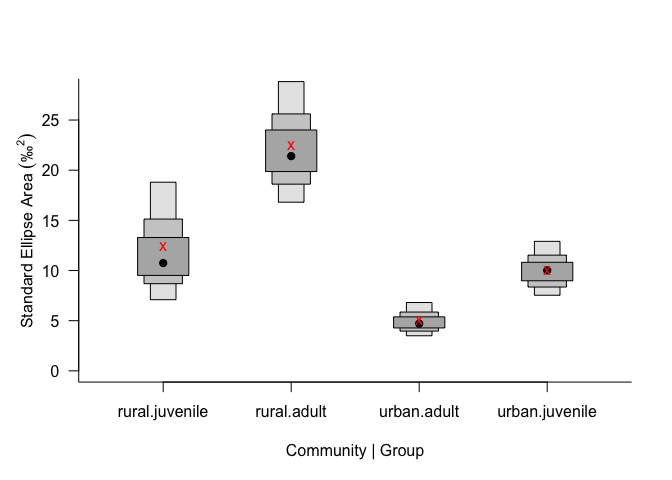


**Fig. S7** Density plot showing Bayesian standard ellipse area (SEA) estimates for each group (i.e., adult and juvenile) and community (i.e., urban and rural). The red crosses indicate SEA estimates corrected for small sample size (SEAc)
